# Oxygen‐Independent Sulfate Radical for Stimuli‐Responsive Tumor Nanotherapy

**DOI:** 10.1002/advs.202200974

**Published:** 2022-04-30

**Authors:** Dandan Ding, Zihan Mei, Hui Huang, Wei Feng, Liang Chen, Yu Chen, Jianqiao Zhou

**Affiliations:** ^1^ Department of Ultrasound Ruijin Hospital Shanghai Jiaotong University School of Medicine Shanghai 200025 P. R. China; ^2^ Shanghai Engineering Research Center of Organ Repair Materdicine Lab School of Life Sciences Shanghai University Shanghai 200444 P. R. China; ^3^ School of Medicine Shanghai University Shanghai 200444 P. R. China; ^4^ Wenzhou Institute of Shanghai University Wenzhou 325000 P. R. China

**Keywords:** melanoma, reactive oxygen species (ROS), skin tissue healing, stimuli‐responsive, ulfate radical

## Abstract

Variant modalities are quested and merged into the tumor nanotherapy by leveraging the excitation from external or intratumoral incentives. However, the ubiquitous hypoxia and the insufficient content of hydrogen peroxide (H_2_O_2_) in tumor microenvironments inevitably hinder the effective production of reactive oxygen species (ROS). To radically extricate from the shackles, peroxymonosulfate (PMS: HSO_5_^−^)‐loaded hollow mesoporous copper sulfide (CuS) nanoparticles (NPs) are prepared as the distinct ROS donors for sulfate radical (•SO_4_^−^)‐mediated and stimuli‐responsive tumor nanotherapy in an oxygen‐independent manner. In this therapeutic modality, the second near‐infrared laser irradiation, together with the released copper ions as well as the heat produced by CuS after illumination, work together to activate PMS thus triply ensuring the copious production of •SO_4_^−^. Different from conventional ROS, the emergence of •SO_4_^−^, possessing a longer half‐life and more rapid reaction, is independent of the oxygen (O_2_) and H_2_O_2_ content within the tumor. In addition, this engineered nanosystem also exerts the function of photoacoustic imaging and skin restoration on the corresponding animal models. This study reveals the enormous potential of sulfate radical in oncotherapy and broadens pave for exploring the application of multifunctional and stimuli‐responsive nanosystems in biomedicine.

## Graphical abstract

The CuS@PMS nanosystems with multiple theranostic functions are maneuverable in stimuli‐responsive sulfate radical‐mediated nanotherapy against melanoma in an oxygen‐independent manner, competent in photoacoustic imaging and precipitating skin regeneration. The high‐performance sulfate radical tumor therapy is implemented via exploiting the outstanding oxidation property of sulfate radical and inducing synergistic activation by photothermal effect and copper ions.

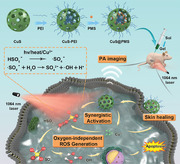

## 1 Introduction

Melanoma is an exceedingly aggressive skin tumor characterized by early metastasis, high recurrence, and low survival rates. To make matters worse, the incidence of melanoma has continued to ascend worldwide in recent years, resulting in the highest mortality rate among cutaneous tumors.^[^ ^1^ ^]^ Currently, the main therapeutic options for melanoma include surgery, radiotherapy, chemotherapy, immunotherapy, and targeted therapy.^[^ ^2^ ^]^ However, the tumor size and location, antitumor drug resistance and adverse side effects impede the effectiveness of the above treatments.^[^ ^3^ ^]^ Therefore, the development of new strategies is in urgent demand to surmount the constraints of clinical melanoma treatment.

Attributing to the merits of precision and controllability, the stimuli‐responsive modalities have been dexterously explored in tumor nanotherapy to maximize treatment benefit and remit systemic toxicity, mainly including the typical photodynamic therapy (PDT), sonodynamic therapy (SDT), and chemodynamic therapy (CDT).^[^ ^4^ ^]^ In these diverse treatments, the excitation from external or intratumoral incentives has been applied to nanomedicine, eventually increasing the level of reactive oxygen species (ROS) and entraining corresponding biological effects (e.g., apoptosis, necrosis, pyroptosis, ferroptosis, autophagy, etc.).^[^ ^5^ ^]^ Despite the superior strategy, stimuli‐responsive nanotherapy still encounters its bottlenecks. One prime smasher is the rigorous reaction conditions which make homogeneous therapeutic regimen incompetent to enable sufficient ROS yield.^[^ ^6^ ^]^ Furthermore, the unique tumor microenvironments (TME), such as the ubiquitous hypoxia in solid tumors, the inadequate content of endogenous H_2_O_2_ and the rapid scavenge of the as‐generated ROS, extremely confine the potency of stimuli‐responsive nanotherapy and restrict their further biomedical applications.^[^ ^7^ ^]^ Encouragingly, with the advent of sulfate radical (•SO_4_^−^) very recently, the dilemma of tumor nanotherapy may be readily solved in an oxygen‐independent manner. Featuring a longer half‐life, more rapid reaction and higher selectivity, •SO_4_^−^ is a more toxic ROS in comparison with other representative types of ROS, such as hydroxyl radical (•OH), and has gained ever‐increasing attention due to their excellent oxidation capability.^[^ ^8^ ^]^ Generally, the production of •SO_4_^−^‐relevant ROS can be implemented through the activation of peroxodisulfate (PDS: S_2_O_8_^−^) and peroxymonosulfate (PMS: HSO_5_^−^) by light, heat and metal ions (ferric, cobalt, copper) in the pH range of 2–9. Importantly, none of the above processes depend on the participation of H_2_O_2_ or O_2_.^[^ ^9^ ^]^ By comparison of the two sources of sulfate radical, PMS is considered to be more easily activated than PDS, on account of its asymmetric structure.^[^ ^10^ ^]^ As an avant‐garde paradigm, phospholipid‐coated Na_2_S_2_O_8_ NPs were constructed, which could generate ROS (•SO_4_^−^ and •OH) irrespective of the amount of H_2_O_2_, O_2_, and pH value.^[^ ^11^ ^]^ However, sulfate radical is still rarely studied in the field of cancer nanotherapy.

To select an appropriate conveyance for establishing a stimuli‐responsive system, we paid heed to copper sulfide (CuS) NPs. As the typical p‐type semiconductor, omnifarious CuS NPs with different sizes and shapes were synthesized since their excellent photothermal properties award them momentous roles in photodynamic and photothermal treatment.^[^ ^12^ ^]^ Especially, the hollow mesoporous CuS NPs feature an interior cavity and abundant mesopores, which enables them as transport agents and endows them with more potency in combination therapy with laser.^[^ ^13^ ^]^ For phototherapy, light in the second near‐infrared (NIR‐II) window (1000‐1700 nm) allows for the deeper tissue penetration due to its reduced tissue scattering and decreased interference by proteins compared to the light in the first near‐infrared (NIR‐I) window (700–1000 nm).^[^ ^14^ ^]^ With higher maximum permissible exposure and better spatial resolution for photoacoustic (PA) imaging, NIR‐II laser further aggrandizes the efficacy of light‐response nanomedicine in tumor theranostics.^[^ ^15^ ^]^ The NIR‐II laser irradiation combined with CuS NPs can induce efficient tumor ablation, followed by the release of copper ions from CuS NPs.^[^ ^16^ ^]^ Compared to ferric ions and cobalt ions, copper ions possess a more stable reaction rate and a higher safety profile in the provocative process of sulfate radical.^[^ ^17^ ^]^ More importantly, the photothermal treatment often leads to therapeutic skin damage,^[^ ^18^ ^]^ while copper ions are capable of promoting angiogenesis,^[^ ^19^ ^]^ facilitating maturation of extracellular matrix (ECM) and thus accelerating tissue healing.^[^ ^20^ ^]^ Considering all these factors, it is a judicious choice to use CuS NPs in stimuli‐responsive modalities for achieving the oxygen‐independent sulfate radical nanotherapy.

**Scheme 1:**
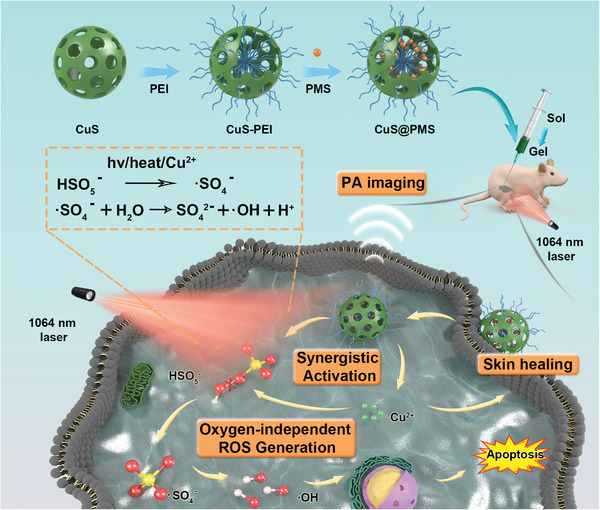
Schematic illustration for the fabrication and theranostic functions of CuS@PMS‐Gel used for photothermal‐enhanced sulfate radical nanotherapy of melanoma, including multiple activation (light, heat and copper ions) of sulfate radical proceeding oxygen‐independent therapy against tumor, PA guidance prior to cancer therapy, and restoration of therapeutic skin injury.

In this work, we rationally engineered PMS‐loaded hollow mesoporous CuS NPs with polyethyleneimine (PEI) modification (designated as CuS@PMS) for •SO_4_^−^‐mediated and stimuli‐responsive tumor nanotherapy in an oxygen‐independent manner. When the CuS@PMS nanosystems enter the tumor cells, the copper ions will be initially released from CuS with the irradiation of 1064 nm, followed by the effective activation of PMS to generate toxic •SO_4_^−^ and •OH, which is independent of the O_2_ and H_2_O_2_ content within the tumor. By designing the multiple roles of CuS in this procedure, the nanosystem is operated effectively and intelligently. The versatile CuS shoulders the responsibility to serve as a carrier for loading PMS and an instructor to monitor the procedure by PA imaging‐guided tumor treatment. Concurrently, the heat produced by CuS after illumination, together with the released copper ions as well as the NIR‐II laser irradiation, work together to activate PMS thus triply ensuring efficient ROS production. Additionally, the released copper ions are capable of promoting skin restoration of therapeutic injuries (**Scheme** **1**). This study introduces the distinct oxygen‐independent sulfate radical for stimuli‐responsive tumor nanotherapy, which provides an alternative but efficient therapeutic paradigm for melanoma treatment.

## 2 Results and Discussion

### 2.1 Fabrication and Characterization of CuS, CuS‐PEI, and CuS@PMS

**Figure 1:**
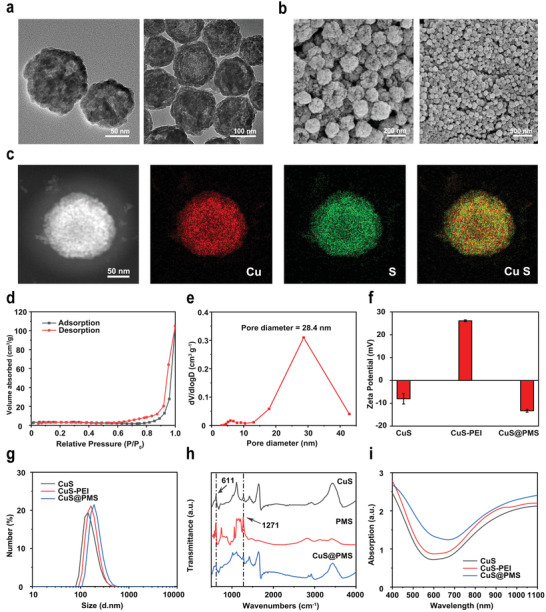
Fabrication and characterization of CuS, CuS‐PEI, and CuS@PMS. a) TEM and b) SEM images of CuS NPs at different magnifications, and c) corresponding elemental mapping images of CuS NPs. d) N_2_ absorption–desorption isotherm and e) pore‐size distribution of CuS NPs. f) Zeta potential and g) hydrodynamic diameters of CuS, CuS‐PEI, and CuS@PMS NPs. Data are expressed as mean ± SD (*n* = 3). h) FTIR spectra of CuS, PMS, and CuS@PMS. i) UV–vis–NIR absorption spectra of CuS, CuS‐PEI, and CuS@PMS NPs.

The CuS NPs were fabricated by using a facile two‐step synthesis method reported previously.^[^ ^21^ ^]^ As observed from the transmission electron microscope (TEM) and scanning electron microscope (SEM) images, the as‐prepared CuS NPs exhibit a uniform spherical morphology with hollow cavities and the average diameter is around 150 nm (**Figure** **1**a,b). The energy dispersive X‐ray spectroscopy (EDS) element mapping also clearly demonstrates the uniform distribution of Cu and S elements in the hollow CuS structure (Figure 1c). The specific surface area of CuS NPs was determined by using the typical N_2_ adsorption/desorption method, and the isotherms as shown in Figure 1d confirm the hollow and mesoporous structure of the obtained CuS NPs. In addition, the specific surface area and pore size of CuS NPs are 22.88 m^2^ g^−1^ and 28.4 nm, respectively (Figure 1e), which facilitates the efficient loading of PMS. After ascertaining the unique structure of CuS NPs, their surface was modified by PEI for generating the positively charged surface, which was followed by constructing CuS@PMS nanosystems through loading PMS into CuS‐PEI by electrostatic interaction. TEM and SEM results display that the morphology and structure of CuS@PMS are resembled with that of CuS NPs (Figure S1, Supporting Information) As shown in Figure 1f, the zeta potential of CuS, CuS‐PEI, and CuS@PMS were determined to be −8.04, 26.1, and −13.3 mV, respectively. According to dynamic light scattering (DLS) measurement, the average hydrodynamic size of CuS NPs is around 200 nm (Figure 1g), which is consistent with the TEM and SEM results. Additionally, the average hydrodynamic size of CuS‐PEI and CuS@PMS are about 209.5 and 244.2 nm, respectively. We further performed Fourier transform infrared (FTIR) analysis to verify the efficient PMS encapsulation (Figure 1h). The wavenumber at 611 cm^−1^ of CuS@PMS corresponds to the Cu—S characteristic peak in CuS and the peak at 1271 cm^−1^ corresponds to the S—O stretching vibration of HSO_5_^−^.^[^ ^22^ ^]^ By ultraviolet–visible–NIR (UV–vis–NIR) spectroscopy, we observe that both CuS@PMS and CuS unfold intense absorption in the NIR‐II region, indicating that CuS@PMS features high photothermal conversion efficiency (Figure 1i). In addition, the content of PMS in CuS@PMS solution (Cu concentration of 50 µg mL^−1^) is determined and calculated to be 10 µg mL^−1^.

### 2.2 In Vitro ROS Production and Copper Ions Release of CuS@PMS

**Figure 2:**
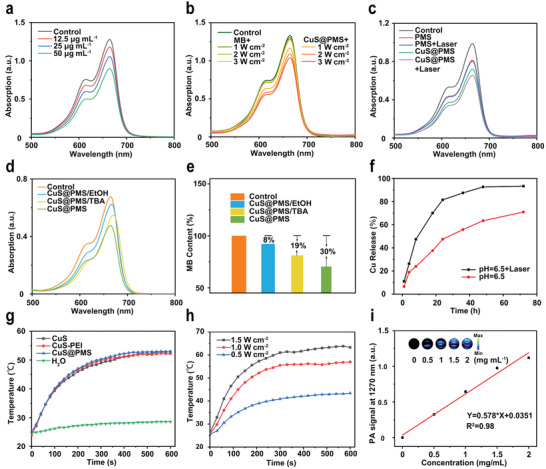
In vitro performance evaluation of CuS@PMS. a) Degradation of MB in the presence of different concentrations of CuS@PMS. b) Degradation of MB upon 1064 nm laser irradiation at various power densities. c) Degradation of MB under different conditions. d,e) ROS capture experiment: EtOH as a scavenger of •SO_4_^−^ and •OH, TBA as a scavenger of •OH. f) Release profiles of copper ions in PBS (pH = 6.5) with or without NIR‐II irradiation. g) Temperature variation of H_2_O, CuS, CuS‐PEI, and CuS@PMS under laser irradiation (1064 nm, 1 W cm^−2^, 10 min). h) Temperature changes of CuS@PMS upon 1064 nm laser irradiation with different power densities. i) The fitted relationship between PA values at 1270 nm with different concentrations of CuS@PMS (0, 0.5, 1, 1.5, 2 mg mL^−1^).

The methylene blue (MB) degradation assay was used for evaluating the capability of CuS@PMS to produce sulfate radical. As shown in **Figure** **2**a, the absorption peak of MB decreases with the increase of CuS@PMS concentration, indicating that PMS promotes the degradation of MB. The effect of laser (NIR‐II, 1064 nm) with varied power densities on the oxidation capability of CuS@PMS was further investigated, and the laser with higher power density activates HSO_5_^−^ to generate more free radicals (Figure 2b). Subsequently, to verify that the photothermal effect and copper ions can synergistically activate HSO_5_^−^ for the enhanced oxidation effect, we compared the MB degradation capacity of PMS and CuS@PMS with or without 1064 nm laser irradiation, respectively (Figure 2c). As expected, the highest sulfate radical yield is observed in the CuS@PMS with laser irradiation group, demonstrating that the photothermal effect and copper ions can effectively activate HSO_5_^−^ for efficient sulfate radical production. We further applied ethanol (EtOH) as a quencher of •SO_4_^−^ and •OH, and exploited *tert*‐butanol (TBA) as a trapping agent of •OH. As shown in Figure 2d,e, the degradation rate of MB was diminished from 30% to 19% with the addition of TBA, while the addition of ethanol resulted in only 8% degradation efficiency of MB, indicating that CuS@PMS can indeed produce •SO_4_^−^ and •OH. Furthermore, we examined the effect of laser on the release of copper ions using inductively coupled plasma‐optical emission spectrometry (ICP‐OES) (Figure 2f), demonstrating that the NIR‐II laser irradiation is capable of accelerating the release of copper ions in an acidic environment, thus doubly promoting the HSO_5_^−^ activation.

### 2.3 In Vitro Photothermal Conversion and PA Imaging of CuS@PMS

To explore the photothermal conversion performance of CuS@PMS nanosystems, the photothermal properties of CuS, CuS‐PEI and CuS@PMS were initially studied, which display similar temperature distributions, indicating that the PMS loading has a negligible effect on the photothermal effect of CuS NPs (Figure 2g). In addition, the temperature increase of CuS@PMS is concentration‐dependent (Figure S2a, Supporting Information). Under 1064 nm laser irradiation at a power density of 1 W cm^−2^, the temperature of CuS@PMS increases rapidly to 53 °C, whereas the temperature of water increases by only 4 °C. The temperature of CuS@PMS varied from 43.4 °C (0.5 W cm^−2^) to 63.6 °C (1.5 W cm^−2^) by adjusting the power density of the NIR‐II laser (Figure 2h). Under NIR‐II laser irradiation, the photothermal performance of CuS@PMS is not altered significantly after four times heating and cooling cycles, confirming the favorable photothermal stability of CuS@PMS (Figure S2b, Supporting Information). Furthermore, the photothermal conversion efficiency of CuS is calculated to be 22.78% via the linear regression curve of the cooling stage after irradiation with a 1064 nm laser (Figure S2c, Supporting Information). As indicated in Figure 2i, the PA signal of CuS@PMS at 1270 nm linearly enhances with the elevating concentration, validating the excellent PA imaging ability of CuS@PMS in the NIR‐II region.

### 2.4 In Vitro Cellular Uptake, Cytotoxicity, and Antitumor Effect of CuS@PMS

**Figure 3:**
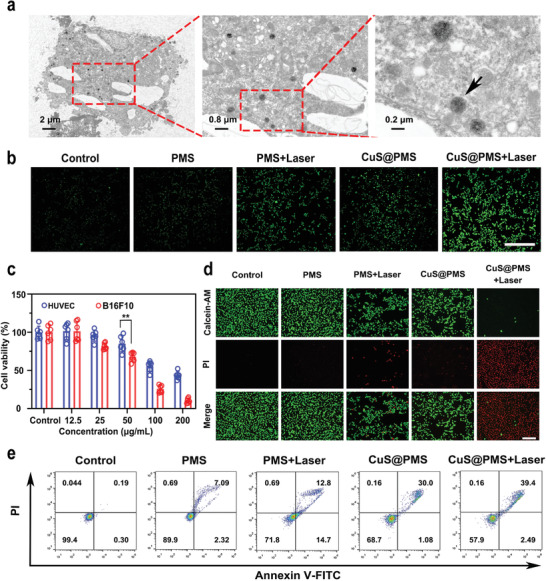
In vitro cellular uptake, cytotoxicity, and antitumor effect. a) Bio‐TEM images of B16F10 cells post co‐incubation with CuS@PMS (50 µg mL^−1^) for 24 h. b) Fluorescence microscopic images of ROS levels in B16F10 cells after various treatments using DCFH‐DA. c) Cell viability of HUVECs and B16F10 cells after incubation with different concentrations of CuS@PMS (0, 12.5, 25, 50, 100, and 200 µg mL^−1^). Data are shown as mean ± SD (*n* = 6, *p*‐values were calculated by two‐way analysis of variance (ANOVA) with Tukey's post hoc test. **p < 0.01). d) Fluorescence microscopy images of B16F10 cells stained by Calcein AM (green, live cells) and PI (red, dead cells) with different treatments. e) FCM analysis of B16F10 cells after different treatments using Annexin V‐FITC and PI. Scale bar: 200 µm.

Prior to investigating the antitumor effect of CuS@PMS nanosystems, it has been found that CuS@PMS could be effectively uptaken by B16F10 cancer cells and mainly distributed in the cytoplasm based on the bio‐TEM observation (**Figure** **3**a). The intracellular ROS levels were measured by 2', 7'‐dichloro‐fluorescein diacetate (DCFH‐DA) to verify the generation of free radicals in vitro. The prominent fluorescent brightness can be observed in CuS@PMS with NIR‐II irradiation group, whereas other groups display faint fluorescent intensity in Figure 3b. Compared with the control group, the relative fluorescence intensities of the other treatment groups were 1.45, 3.14, 2.53, and 5.22, respectively, indicating that substantial ROS are produced in the cells after the treatment of CuS@PMS with NIR‐II irradiation. Subsequently, the cytotoxicity of CuS@PMS to Human Umbilical Vein Endothelial Cells (HUVECs) and B16F10 cells was detected by the typical CCK‐8 assay. As can be seen from Figure 3c, after treatment of CuS@PMS for 24 h, the viability of both kinds of cells is decreased with the elevated doses of CuS@PMS. When the concentration of CuS@PMS is more than 50 µg mL^−1^, the viability of tumor cells is significantly lower than that of normal cells. These results manifest that the high concentration of CuS@PMS has cytotoxicity on both HUVECs and B16F10 cells to some extent, whereas B16F10 cells are more sensitive to its cytotoxic effect. In order to appraise the antitumor effect of CuS@PMS in vitro, we stained living and dead cells with Calcein‐AM and propidium iodide (PI), respectively. Compared to cancer cells treated with CuS@PMS only or PMS with NIR‐II irradiation, abundant dead cells were inspected in the CuS@PMS with NIR‐II irradiation group, substantiating that photothermal‐ and copper ions‐activated sulfate radical‐based nanotherapy has induced significant antitumor efficacy (Figure 3d). Furthermore, Annexin V‐FITC and PI staining assay was harnessed to further reveal the therapeutic effect of sulfate radical (Figure 3e) by flow cytometry (FCM). After irradiation with NIR‐II laser for 5 min, CuS@PMS induces 41.89% of cell apoptosis, which distinctly exceeds the CuS@PMS group (31.08%) and PMS with irradiation group (27.5%).

### 2.5 In Vivo PA Imaging and Biocompatibility of CuS@PMS‐Embedded Hydrogel (CuS@PMS‐Gel)

Attributing to the employment of gel that can avoid leakage of NPs during intratumoral injection and facilitate the localized melanoma therapy, CuS@PMS were embedded into an injectable thermosensitive hydrogel (Gel) with biological compatibility to ensure affluent drug accumulation in the tumor area and abate side effects toward normal tissues.^[^^]^ Through SEM detection of Gel and CuS@PMS‐Gel, we can observe profuse pores inside the Gel (Figure S3a,b, Supporting Information). The element mapping of CuS@PMS‐Gel exhibits the homogeneous distribution of Cu and S elements originating from CuS NPs, as well as O, P and Na elements from the Gel (Figure S3c, Supporting Information). In order to survey the injectability under the physiological environment, the hydrogel solution was tardily injected into 37 °C warm water with a syringe and gradually converted to gel (Figure S4a, Supporting Information). We also placed two eppendorf (EP) tubes containing blank Gel and CuS@PMS‐Gel solution, respectively, at 37 °C and inverted them after 5 min, suggesting that the solution has formed the gel and no longer flowed (Figure S4b,c, Supporting Information).

**Figure 4:**
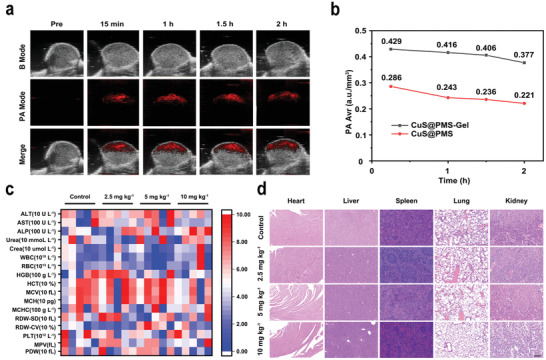
In vivo PA imaging and biocompatibility of CuS@PMS‐Gel. a) PA images in tumor tissues before and after intratumoral injection of CuS@PMS‐Gel. b) The average value of PA signal at tumor regions after varied treatment durations. c) Hematological indexes of the mice after subcutaneous injection with PBS and CuS@PMS‐Gel (2.5, 5, 10 mg kg^−1^) for 30 d. d) HE staining of the major organs from mice subcutaneously injected with PBS and CuS@PMS‐Gel (2.5, 5, 10 mg kg^−1^) after 30 d. Scale bar: 100 µm.

B16F10 tumor‐bearing nude mice were used to evaluate the PA imaging capability of CuS@PMS solution and CuS@PMS‐Gel in vivo. PA signals in the NIR‐II region of the tumor site were monitored at different time intervals after intratumoral injection (**Figure** **4**a and Figure S5, Supporting Information). Compared with the low and diminished PA signals in the tumor region of the mice injected with CuS@PMS solution, the distinct and persistent PA signal in CuS@PMS‐Gel group indicates abundant accumulation of CuS@PMS within the tumor tissues, significantly indicating that the gel can avoid leakage of NPs after intratumoral injection and CuS@PMS‐Gel can serve as a potential PA imaging agent to guide tumor therapy in the NIR‐II region (Figure 4b). To verify its biocompatibility for potential clinical application, twenty healthy mice were subcutaneously injected with saline (Control) and CuS@PMS‐Gel (Cu doses at 2.5, 5, 10 mg kg^−1^). The conventional blood analysis, biochemical index detection and HE staining demonstrate that no obvious inflammation, histopathological changes and other abnormal phenomena are observed in the CuS@PMS‐Gel group for 30 d post‐injection, proving the benign biocompatibility of CuS@PMS‐Gel within the tested dosage (Figure 4c,d).

### 2.6 In Vivo Antitumor Effect of CuS@PMS‐Gel

**Figure 5:**
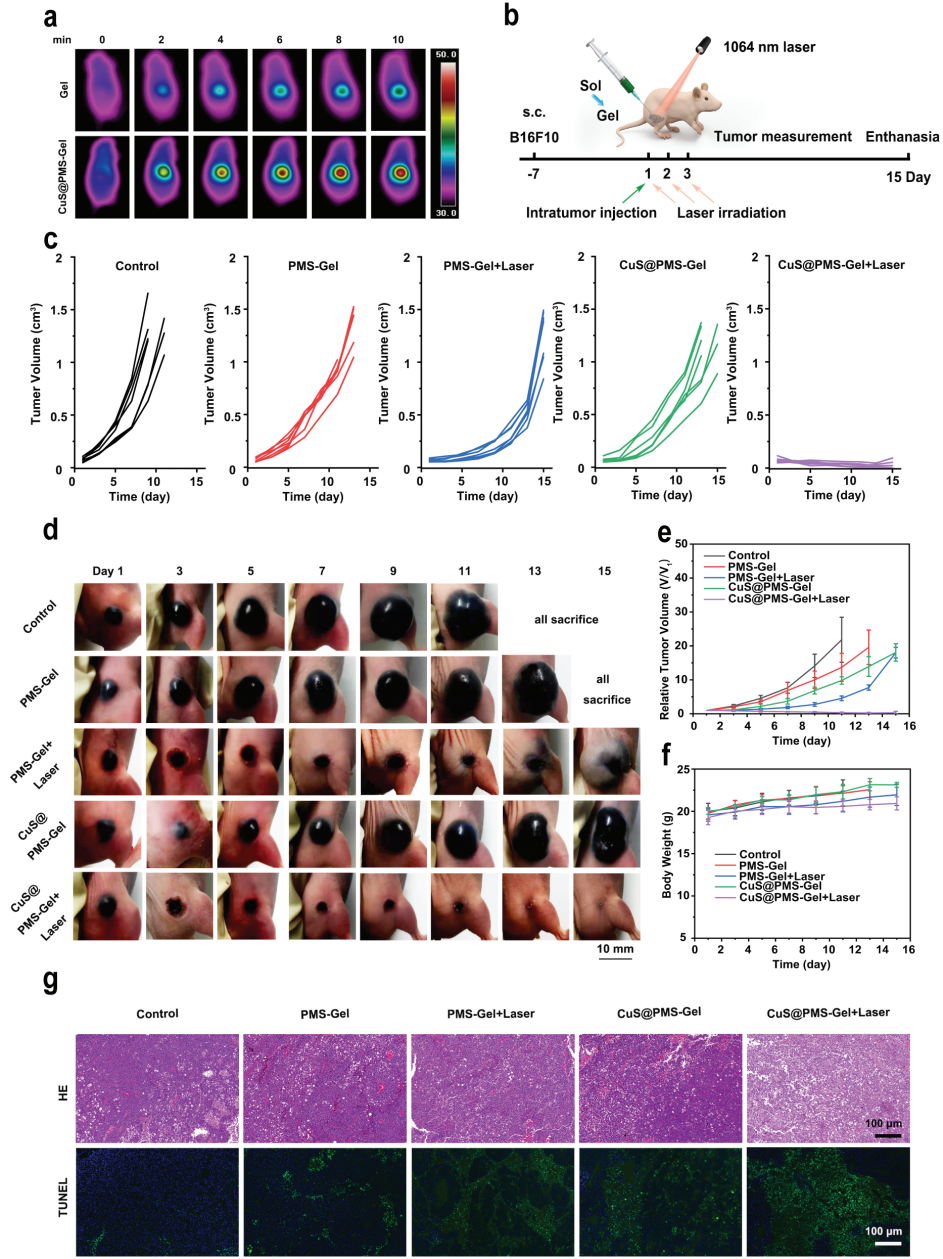
In vivo antitumor effect of CuS@PMS‐Gel. a) IR thermal images of B16F10 tumor‐bearing nude mice with intratumoral injection of Gel or CuS@PMS‐Gel followed by 1064 nm laser irradiation (1 W cm^−2^) at varied time intervals. b) In vivo therapeutic protocol of CuS@PMS‐Gel against melanoma. c) Individual tumor growth curves of mice after various treatments. d) Representative digital pictures of mice during treatments. e) Tumor growth curves and f) body weight curves of mice after different treatments. Data are expressed as mean ± SD. g) HE and TUNEL staining of tumor harvested from mice after various treatments.

Encouraged by the effective in vitro antitumor effect, we further carried out the in vivo antitumor experiment of the engineered CuS@PMS‐Gel. The photothermal imaging of CuS@PMS‐Gel in vivo was initially performed after the intratumor injection of 50 µL Gel and 50 µL CuS@PMS‐Gel (Cu dose at 2.5 mg kg^−1^), respectively. The temperature in tumors of mice injected with CuS@PMS‐Gel increases significantly under 1064 nm laser (1 W cm^−2^) irradiation for 10 min, which reaches 47.8 °C (**Figure** **5**a). In contrast, the local temperature in tumors of mice injected with Gel only rises to 40.1 °C. The aforementioned results signify that CuS@PMS‐Gel exhibits favorable thermogenesis in vivo, which can not only induce photothermal ablation of tumor tissues, but also provide sufficiently high temperature to activate the production of sulfate radical and further improve the therapeutic effect synergistically.

To investigate the in vivo antitumor property of CuS@PMS‐Gel, the B16F10 melanoma tumor‐bearing nude mice were randomly divided into various groups: Control, PMS‐Gel, PMS‐ Gel with NIR‐II laser (1064 nm) irradiation, CuS@PMS‐Gel, CuS@PMS‐Gel with NIR‐II laser irradiation, and the mice in the NIR‐II laser group was irradiated once a day from day 1 to day 3 after intratumoral injection of PMS‐Gel or CuS@PMS‐Gel. The mice were euthanized on the 15th day to end the experiment (Figure 5b). As shown in Figure 5c,d, the tumor volume of the Control group and PMS‐Gel group increases at a great lick, and the mice were all sacrificed at the end of treatment. In contrast, the PMS‐Gel with NIR‐II laser irradiation group and the CuS@PMS‐Gel group manifest a certain degree of tumor growth inhibition at the initial stage of treatment, but it is remarkable that the tumor volume is still increasing promptly at the later stage of treatment. Comparatively, in the CuS@PMS‐Gel with NIR‐II laser irradiation group, the tumor volume remains at an extremely low level within 15 d of monitoring, indicating that the developed sulfate radical‐based nanotherapy activated by copper ion combined with photothermal ablation induces a mighty tumor inhibition effect (Figure 5e). In addition, during the treatment, there is no significant difference in body weight among all groups (Figure 5f), confirming that sulfate radical‐based nanotherapy features desirable therapeutic biosafety. Representative mice photos (Figure S6a, Supporting Information) and tumor photos (Figure S6b, Supporting Information) at the end of different treatments also intuitively reflect the significant therapeutic effect of sulfate radical treatment based on the synergistic activation by copper ion and photothermal effect. The efficacy of each group was further evaluated by HE and TUNEL staining (Figure 5g). The relative positive cell percentages in TUNEL staining of the other treatment groups were 3.73, 9.78, 8.37, and 17.80‐fold higher than that of the control group, respectively. The tumor cells in the CuS@PMS‐Gel with NIR‐II laser irradiation group display obvious apoptosis and necrosis in comparison with the other groups.

### 2.7 Tissue Healing Performance of CuS@PMS‐Gel

**Figure 6:**
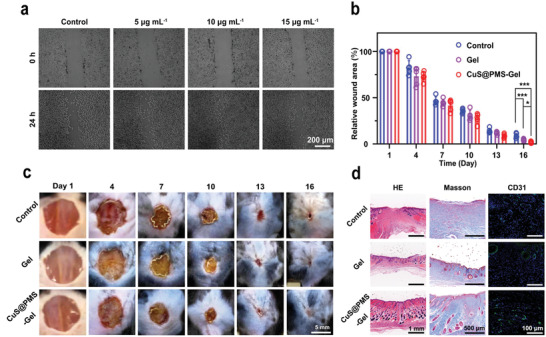
Tissue healing assay. a) In vitro scratch assay of HUVECs cultured with media containing different concentrations of CuS@PMS. b) Relative wound area and c) wound photographs with different treatments. Data are shown as mean ± SD (*n* = 5, *p*‐values were calculated by two‐way analysis of variance (ANOVA) with Tukey's post hoc test. *p < 0.05; ***p < 0.001). d) HE, Masson, and immunofluorescence (blue: nucleus, green: CD31) staining images at the end of therapy.

The effect of CuS@PMS on HUVECs cell migration was investigated by the typical scratch assay (**Figure** **6**a). Compared with the Control group, different concentrations of CuS@PMS could expedite the migration of HUVECs cells. Especially for the 10 µg mL^−1^ group, the HUVECs cells almost cover the scratches after 24 h incubation. To further evaluate the effect of CuS@PMS‐Gel on wound healing, we used a typical diabetic mice model and divided diabetic mice into three groups randomly. As shown in Figure 6b, on the 16th day of treatment, the relative wound area of CuS@PMS‐Gel (1.85%) was much smaller than that of the Control group (8.94%) and Gel group (4.56%), indicating that CuS@PMS‐Gel promotes the wound healing commendably. The palingenetic skin in the CuS@PMS‐Gel group has covered the entire wound surface on the 16th day, while the skin of the Gel and Control groups still exists the obvious gaps (Figure 6c). Furthermore, compared with the Gel and Control groups, the epithelium at the wound site in the CuS@PMS‐Gel group is continuous and exhibits more mature skin accessory organ formation according to the HE images (Figure 6d). Masson and CD31 staining were further performed to reveal the underlying mechanism of CuS@PMS‐Gel for promoting wound healing (Figure 6d). Masson staining was used to display the expression of collagen which plays an important role in tissue remodeling, and CD31 staining was used to assess capillary formation. The positive cell rates of CD31 staining in the Gel group and the CuS@PMS‐Gel group were 0.96‐ and 1.80‐fold as compared to the control group. Staining results present that the CuS@PMS‐Gel group manifests obvious collagen deposition and a high level of CD31 expression in the wound area. It is collectively substantiated that CuS@PMS‐Gel can not only increase collagen expression, but also promote the formation of capillaries, possibly resulting from the release of copper ions.^[^ ^24^ ^]^

## 3 Conclusions

In summary, we herein proposed a distinct sulfate radical‐mediated tumor nanotherapy by manufacturing hollow mesoporous PMS‐loaded CuS (CuS@PMS) nanosystems. Because the generation of sulfate radical in the treatment is oxygen‐independent, this nanoplatform fundamentally gets rid of the predicament that classical ROS‐involved therapy is limited by the scanty content of O_2_ and endogenous H_2_O_2_ in TME, not to mention the splendid reaction characteristics of the generated sulfate radical which are superior to traditional ROS. Simultaneously, the synergistic activation of the intrinsic photothermal effect and copper ions guarantee the generation of sulfate radical for efficient dynamic nanotherapy against melanoma. Additionally, the engineered CuS@PMS nanosystem can serve as a tempting PA imaging agent for implementing imaging‐guided cancer treatment. Benefiting from its excellent PA imaging and outstanding anticancer properties, this engineered multifunctional nanosystem features considerable potential for the diagnosis and treatment of melanoma. Especially, the copper ions released from the CuS@PMS nanosystem can boost the process of skin healing, thereby facilitating the skin repair process after photothermal‐enhanced sulfate radical treatment as well. This work not only broadens the construction and biomedical application of sulfate radical‐involved nanosystems, but also provides a new paradigm for the treatment of melanoma with concurrent antitumor and skin tissue‐engineering performances.

## Conflict of Interest

The authors declare no conflict of interest.

## Supporting information

Supporting Information

## Data Availability

The data that support the findings of this study are available from the corresponding author upon reasonable request.
