# Supplementary material for: Oxygen‐Independent Sulfate Radical for Stimuli‐Responsive Tumor Nanotherapy
Source: Adv Sci (Weinh). 2022 Apr 30;9(17):2200974. doi: 10.1002/advs.202200974 (PMC9189647; doi:10.1002/advs.202200974)
Supplement: Supplementary file 1 — Supporting Information [file ADVS-9-2200974-s001.pdf]

ADVANCED SCIENCE
Open Access

Supporting Information

for *Adv. Sci.*, DOI 10.1002/advs.202200974

Oxygen-Independent Sulfate Radical for Stimuli-Responsive Tumor Nanotherapy

Dandan Ding, Zihan Mei, Hui Huang, Wei Feng, Liang Chen, Yu Chen* and Jianqiao Zhou**

Supporting Information

Oxygen-Independent Sulfate Radical for Stimuli-Responsive Tumor Nanotherapy

Dandan Ding, Zihan Mei, Hui Huang, Wei Feng, Liang Chen, Yu Chen*and Jianqiao Zhou**

D. Ding, Z. Mei, Prof. J. Zhou
Department of Ultrasound, Ruijin Hospital, Shanghai Jiaotong University School of Medicine
Shanghai 200025 P. R. China
E-mail: zjq11432@rjh.com.cn (J. Zhou)

H. Huang, Dr. L. Chen, A/Prof. W. Feng, Prof. Y. Chen
Shanghai Engineering Research Center of Organ Repair, Materdicine Lab, School of Life Sciences, Shanghai University
Shanghai 200444, P. R. China
E-mail: huanghuish@shu.edu.cn (H. Huang), chenyuedu@shu.edu.cn (Y. Chen)

Prof. Y. Chen
School of Medicine, Shanghai University, Shanghai, 200444, P. R. China.

Prof. Y. Chen
Wenzhou Institute of Shanghai University, Wenzhou, 325000, P. R. China.

A: Experimental Section

Materials: Copper (II) chloride dihydrate ($CuCl_2·2H_2O$), sodium sulfide nonahydrate ($Na_2S·9H_2O$), polyethyleneimine (PEI), peroxymonosulfate (PMS)/Oxone® ($KHSO_5·0.5KHSO_4·0.5K_2SO_4$), methylene blue (MB) and chitosan were purchased from Aladdin Chemicals Co., Ltd. (Shanghai, China). Poly(vinylpyrrolidone) (PVP-K40) and streptozocin were acquired from Sigma-Aldrich. Hydrazine hydrate aqueous solution ($N_2H_4·H_2O$) was obtained from Sinopharm Chemical Reagent Co., Ltd. (China). Acetic acid, β-Glycerophosphate(β-GP) and tert-butyl alcohol (TBA) were acquired from Shanghai Macklin Biochemical Co., Ltd.

Fabrication of HCuSNPs (CuS), PEI-modified HCuSNPs (CuS-PEI) and PMS-loaded, PEI-modified HCuSNPs (CuS@PMS): CuS NPs were synthesized conforming to the literature.[1] Firstly, 100 μL of $CuCl_2$ solution (67.5 mg mL^{-1}), 0.24 g of PVP-K40 and 25 mL of NaOH (pH = 9) solution were added in sequence to 25 mL of deionized (DI) water under magnetic stirring. Afterward, 6.4 μL of hydrazine anhydrous solution (85%) was transferred to the mixture, followed by the addition of 200 μL of Na_2S aqueous solution (320 mg mL^{-1}). The reaction was calefied at 60 °C for 2 h. CuS NPs were obtained after centrifugation at 11,000 rpm for 10 min and washing with DI water. To obtain CuS-PEI NPs, 20 mL of PEI (1 mg mL^{-1}) was added to 20 mL of CuS (1 mg mL^{-1}). The mixture was stirred for 6 h and washed three times to collect CuS-PEI. By mixing PMS with CuS-PEI solution (1:1, w/w) for 60 min, CuS@PMS was obtained after centrifugation at 11,000 rpm for 5 min and washing four times with DI water.

Characterization: Transmission electron microscopy (TEM) photographs and element mapping were acquired on a JEM-2100F electron microscope (200 kV). Scanning electron microscopy (SEM) images were recorded on a ZEISS EVO 15 microscope (Oxford instrument, UK). Brunner-Emmet-Teller (BET) analysis of CuS was conducted by a Belsorp-max specific surface area and porosity analyzer. Size and zeta potential measurements were obtained on a Zeta sizer Nano series (Nano ZS90, Malvern Instrument Ltd.). Fourier transformed infrared (FTIR) spectra were obtained by using a FTIR spectrometer (IRAffinity-1S, Shimadzu Company, Japan). Ultraviolet-Visible-NIR (UV-vis-NIR) absorption spectra of nanoparticles were conducted by a UV-3101PC Shimadzu spectrometer. The element quantitative analysis of the sample was determined by inductively coupled plasma-optical emission spectrometry (Agilent 730, Agilent Technologies, US). A Vevo LAZR-X PA imaging system (Fujifilm VisualSonics Company) was harnessed to acquire photoacoustic images. The concentration of Cu was measured by inductively coupled plasma-optical emission spectrometer (ICP-OES, ICP-OES730, Agilent, USA).

In Vitro MB Degradation Evaluation: Different concentrations of CuS@PMS (Cu concentration at 0, 12.5, 25, 50 μg mL^{-1}) were added to the MB solution (0.125 mg mL^{-1}) and were irradiated with a 1064 nm laser for 5 min (1 W cm^{-2}), as well as the absorption was detected after 60 min. Then, CuS@PMS (Cu concentration at 12.5 μg mL^{-1}) was mixed with MB solution and irradiated with a 1064 nm laser (1, 2 and 3 W cm^{-2}) for 5 min and the absorption was detected after 60 min. Similarly, the production of ROS was evaluated after the following different

treatments: (1) Control, (2) PMS (10 μg mL^{-1}), (3) PMS with irradiation (1064 nm, 1 W cm^{-2}, 5 min), (4) CuS@PMS (Cu concentration at 50 μg mL^{-1}), (5) CuS@PMS with irradiation (1064 nm, 1 W cm^{-2}, 5 min). For ROS capture experiment, TBA was leveraged as a quencher for •OH, while Ethanol (EtOH) was utilized as a quencher for $\bullet SO_4^-$ and •OH.[2]

Bio-TEM Observation: To observe the cell endocytosis, the B16F10 cells were incubated with CuS@PMS-containing culture medium (Cu concentration at 50 μg mL^{-1}) for 24 h. Then, the cells were fixed in 2.5% glutaraldehyde for 5 min, followed by scrape and centrifugation (1000 rpm, 3 min). The collected cells were finally fixed with glutaraldehyde and sliced for bio-TEM observation.

Intracellular ROS Production: B16F10 cells were planted into 6-well plates (1×10^5 cells per well) and incubated for 24 h. Then, the cells were co-incubated with PMS (10 μg mL^{-1}) and CuS@PMS (Cu concentration at 50 μg mL^{-1}) for 4 h. Laser irradiation (1064 nm, 1 W cm^{-2}, 5 min) was conducted after that. DCFH-DA was added to the above dishes and co-incubated for 30 min. Finally, the intracellular fluorescence intensity of DCF was observed by a fluorescence microscope to evaluate the production of ROS. The fluorescence semi-quantitative data were analyzed by Image J software.

In Vitro Cytotoxicity: Human Umbilical Vein Endothelial Cells (HUVECs) and B16F10 cells were seeded in 96-well culture plates (5×10^3 cells per well) and cultured for 24 h. Subsequently,

100 mL of fresh culture medium containing CuS@PMS with different Cu concentrations (0, 12.5, 25, 50, 100 and 200 μg mL^{-1}) superseded the original medium. After 24 h of incubation, the standard cell counting kit-8 (CCK-8) assay was used to assess the viability of cells (n = 6).

In Vitro Antitumor Performance: B16F10 cells were planted into 6-well plates with a density of 1×10^5 cells per well and incubated for 24 h to proceed live–dead cell staining assay. Medium containing PMS (10 μg mL^{-1}) and CuS@PMS (Cu concentration at 50 μg mL^{-1}) was used to replace the original medium. For the treatment with laser irradiation, the cells were exposed to a 1064 nm laser (1 W cm^{-2}, 5 min). After another 18 h of incubation, the cells were washed with PBS and stained by 100 μL of the solution of Calcein-AM (1 μM) and propidium iodide (PI) (5 μM) for 15 min. The fluorescence intensity was recorded by a fluorescence microscope.

For apoptosis analysis by flow cytometry (FCM), B16F10 cells were co-incubated with PMS (10 μg mL^{-1}) and CuS@PMS (Cu concentration at 50 μg mL^{-1}) after they adhered to the wall of the dish overnight. Followed by 5 min of a 1064 nm laser irradiation (1 W cm^{-2}) and another 18 h of incubation, the cells were trypsinized and washed with PBS. Then, the Annexin V (5 μL) and PI (5 μL) were added to distinguish cells in different apoptotic states. Finally, the apoptosis condition of different groups was determined by a flow cytometer.

Preparation of Injectable CuS@PMS-embedded Hydrogel (CuS@PMS-Gel): The injectable thermogel was synthesized according to the previous literature.[3] By gradually adding 100 mg chitosan to 4 mL acetic acid (0.1 M) under continuous stirring until the solution was clear. Then, 600 mg β-GP was dissolved in 1 mL deionized water. The two solutions were stored at 4 °C for

20 min. After that, the β-GP solution was added dropwise into the chitosan solution under gently stirring in the ice bath. After the formation of a homogeneous liquid solution, injectable PMS-embedded thermogel (PMS-Gel) and CuS@PMS-Gel were prepared by adding PMS and CuS@PMS respectively into blank hydrogel with the volume ratio of 1:9 and mixed for 2 min.

Animal Experiments: Healthy female ICR mice (5 weeks), female BALB/c nude mice (5 weeks) and male C57BL/6 mice (5-6 weeks) were purchased from Jiangsu GemPharmatech Co., Ltd. All the animal procedures were carried out under the approval of ethics by Ethics Committee of Shanghai University.

Photoacoustic (PA) Imaging Capacity of CuS@PMS-Gel In Vivo: 5-weeks-old female Balb/c nude mice bearing B16F10 tumor were used to evaluate the PA performance of CuS@PMS-Gel *in vivo*. When the tumor volume reached around 300 mm^3, the PA images of the mice were acquired before injection and at different time points (15 min, 1 h, 1.5 h and 2 h) after intratumoral injection of CuS@PMS solution and CuS@PMS-Gel (Cu concentration at 10 mg kg^{-1}).

Biosafety Evaluation of CuS@PMS-Gel In Vivo: Twenty healthy 5-weeks-old female healthy ICR mice were randomly divided into four groups (n = 5) and injected with saline (Control) and CuS@PMS-Gel (Cu concentration at 2.5, 5 and 10 mg kg^{-1}) respectively. After 30 days, the blood samples were collected for conventional blood analysis and serum biochemistry test. Then, the mice were sacrificed and the main organs (heart, liver, spleen, lung and kidneys) were

harvested for further histological analysis through hematoxylin and eosin (HE) staining.

In Vivo Photothermal Performance of CuS@PMS-Gel: Gel (50 μL) and CuS@PMS-Gel (50 μL, Cu concentration at 2.5 mg kg^{-1}) were injected intratumorally into female tumor-bearing mice. After injection, tumors were irradiated with a 1064 nm laser (1 W cm^{-2}, 10 min). An IR thermal camera was used to monitor the temperature of tumors and record IR thermal images.

In Vivo Melanoma Tumor Growth Inhibition: B16F10 cells (1×10^6) were injected subcutaneously into the right flank of female Balb/c nude mice (5 weeks old) to establish the melanoma tumor models. When the tumor volume reached around 50-100 mm^3, the mice were divided into 5 groups (n = 7): Control, PMS-Gel, PMS-Gel with laser irradiation, CuS@PMS-Gel, CuS@PMS-Gel with laser irradiation. Then, the mice in each group were intratumorally administered with 50 μL PMS-Gel (0.5 mg kg^{-1}) and CuS@PMS-Gel (Cu concentration at 2.5 mg kg^{-1}) except for the Control group. For the laser treatment groups, the mice were treated with a 1064 nm laser irradiation (1 W cm^{-2}, 10 min) once a day from day 1 to day 3 after injection. The tumor volume and body weight of mice were measured every 2 days. The tumor volume (V) was calculated by a formula of $V= (ab^2)/2$, where a and b refer to the largest length and width of tumors. Relative tumor volumes were calculated by V_t/V_1, while V_1 and V_t represent V on day 1 and the other different days (3, 5, 7, 9, 11, 13 and 15), respectively. 15 days later, the mice were euthanized and the tumors were collected for further staining and analysis. The fluorescence semi-quantitative data were analyzed by Image J software.

Scratch Assay: HUVECs were planted on 6-well plates with a density of 3×10^5 cells per well and cultured for 24 h. The confluent cell monolayer was scratched vertically by a 200-μL pipet tip and different concentrations of CuS@PMS were added respectively. An inverted microscope was used for observation after 24 h incubation.

In Vivo Chronic Wound Healing: To establish diabetic mice models, C57BL/6 mice (male, 5-6 weeks) were intraperitoneally injected with streptozocin (50 mg kg^{-1}, 0.1 M in citrate buffer solution) per day for 5 days.[4] We monitored the glucose levels of mice every 3 days and diabetic mice were considered to be triumphantly established when blood glucose levels exceeded 20 mmol/L. After a full thickness wound with a diameter of 10 mm was formed, mice were randomized into three groups (n = 5): Control, Gel and CuS@PMS-Gel (Cu concentration at 0.5 mg kg^{-1}). 200 μL of corresponding gels were filled with the skin defects except for the control group. The skin wounds were photographed every three days and the area of the wound was calculated by ImageJ [5]. The relative wound area was calculated according to the following equation: Relative wound area (%) = $W_t/W_1 \times 100$ %. W_1 and W_t indicated the wound area on day 1 and the other different days (day 4, 7, 10, 13 and 16). All mice were sacrificed on day 16. The skin samples were collected and then stained for histological analysis. The fluorescence semi-quantitative data were analyzed by Image J software.

Statistical Analysis: Quantitative data were expressed as the mean ± standard deviation (SD).

Two-way analysis of variance (ANOVA) was employed with Tukey's post hoc test and differences were regarded significant if $p < 0.05$ (*$p < 0.05$, **$p < 0.01$, ***$p < 0.001$). All statistical analyses were performed with GraphPad Prism (8.0).

B: Supplementary Figures

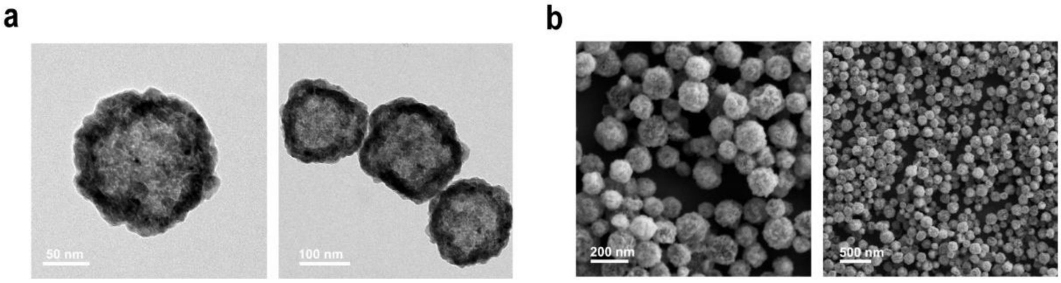

Figure S1. (a) TEM and (b) SEM images of CuS@PMS NPs at different magnifications.

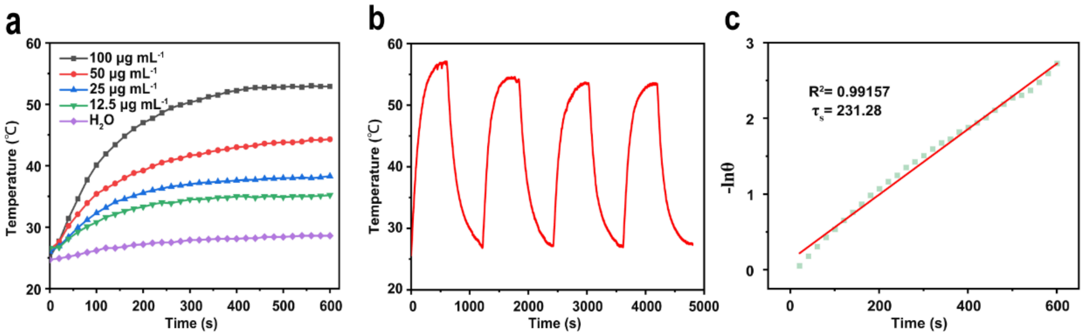

Figure S2. (a) Heating curves of CuS@PMS at different Cu concentrations (12.5, 25, 50 and 100 μg mL^{-1}) as irradiated by 1064 nm laser (1 W cm^{-2}). (b) Heating curve of CuS@PMS for four laser on/off cycles under 1064 nm laser irradiation (1 W cm^{-2}). (c) The linear regression curve and time constant for heat transfer of cooling stage.

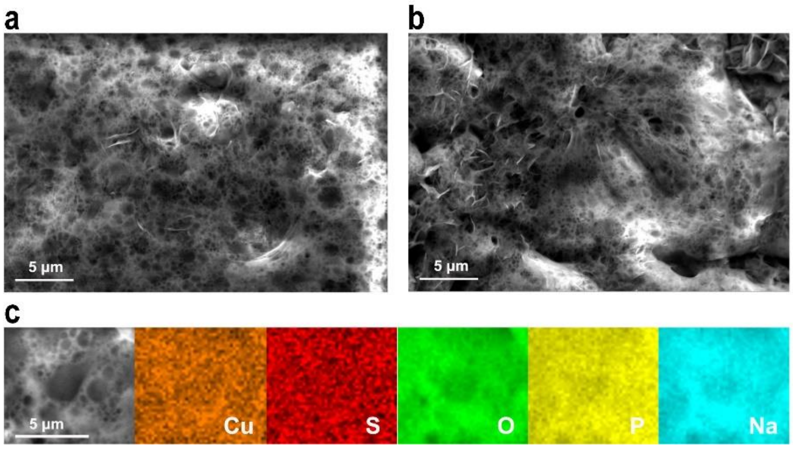

Figure S3. SEM images of (a) Gel and (b) CuS@PMS-Gel. (c) HAADF images and corresponding element mapping images of CuS@PMS-Gel.

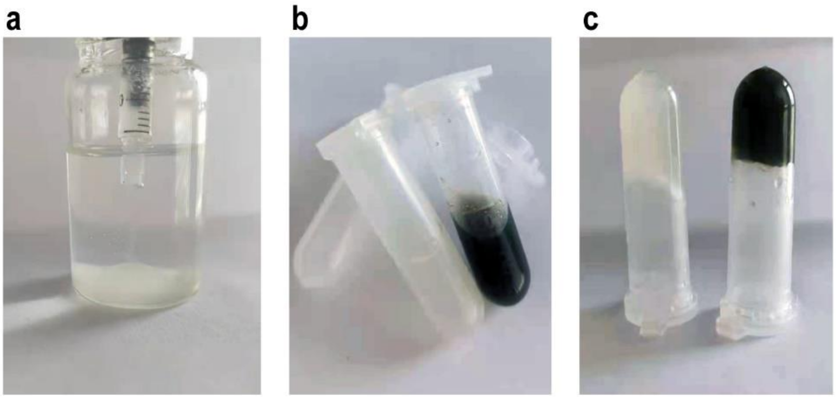

Figure S4. (a) The digital image of Gel solution injected into 37°C warm water with a syringe. Images of Gel and CuS@PMS-Gel before (b) and after (c) placed at 37°C for 5 min.

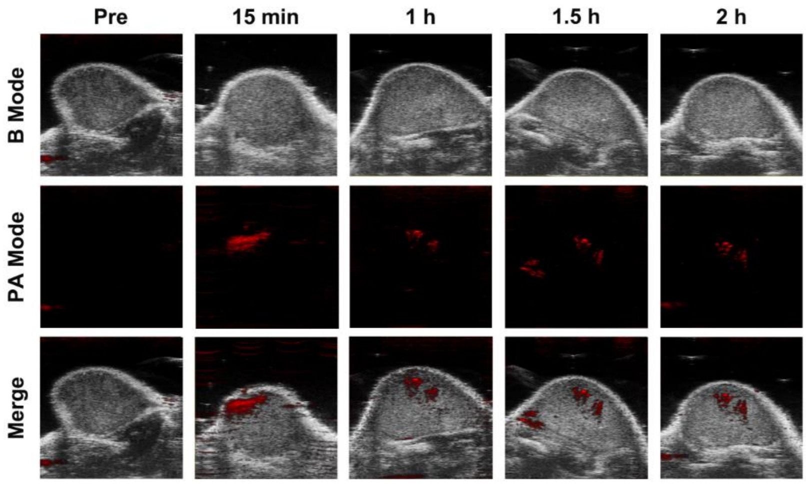

Figure S5. PA images in tumor tissues before and after intratumoral injection of CuS@PMS solution.

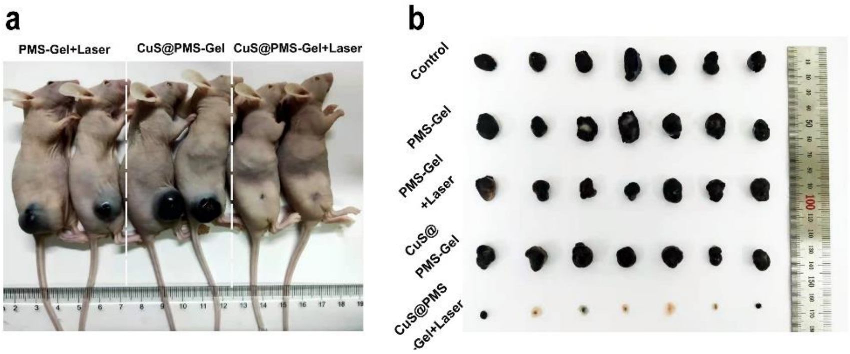

Figure S6. (a) Representative photos of mice on day 15th after different treatments (mice in the Control group and the PMS-Gel group sacrificed before the end of the treatment according to the welfare and ethical requirements of experimental animals). (b) Photographs of dissected tumors after varied treatments.

Reference

[1] a) W. Liu, H. Xiang, M. Tan, Q. Chen, Q. Jiang, L. Yang, Y. Cao, Z. Wang, H. Ran, Y. Chen, *ACS Nano.* **2021**, 15, 6457; b) Q. Li, L. Sun, M. Hou, Q. Chen, R. Yang, L. Zhang, Z. Xu, Y. Kang, P. Xue, *ACS Appl. Mater Interfaces.* **2019**, 11, 417.

[2] Z. Y. Guo, C. X. Li, M. Gao, X. Han, Y. J. Zhang, W. J. Zhang, W. W. Li, *Angew. Chem., Int. Ed.* **2021**, 60, 274.

[3] P. Zhu, Y. Chen, J. Shi, *Adv. Mater.* **2020**, 32, e2001976.

[4] X. Bai, X. Li, J. Tian, L. Xu, J. Wan, Y. Liu, *Free Radic Biol Med.* **2018**, 118, 71.

[5] X. Wang, B. Ma, J. Xue, J. Wu, J. Chang, C. Wu, *Nano Lett.* **2019**, 19, 2138.
